# Survey on Implementation of One Health Approach for MERS-CoV Preparedness and Control in Gulf Cooperation Council and Middle East Countries

**DOI:** 10.3201/eid2503.171702

**Published:** 2019-03

**Authors:** Elmoubasher Abu Baker Farag, Mohamed Nour, Ahmed El Idrissi, Jaouad Berrada, Aya Moustafa, Minahil Mehmood, Mahmoud H. Mahmoud, Ahmed M. El-Sayed, Farhoud Alhajri, Mohammed Al-Hajri, Osama Ahmed Hassan, Hamad Al-Romaihi, Mohamed Al-Thani, Salih A. Al-Marri, Marion P.G. Koopmans, Mohamed Haroun Ismail

**Affiliations:** Ministry of Public Health, Doha, Qatar (E. Abu Baker Farag, M. Nour, A. Moustafa, M. Mehmood, M.H. Mahmoud, A.M. El-Sayed, M. Al-Hajri, H. Al-Romaihi, M. Al-Thani, S.A. Al-Marri);; Food and Agriculture Organization of the United Nations, Rome, Italy (A. El Idrissi);; Institut Agronomique et Vétérinaire Hassan, Rabat, Morocco (J. Berrada);; Ministry of Municipality and Environment, Doha (M.H. Mahmoud, F. Alhajri, M.H. Ismail);; Center for Global Health of Oslo University, Oslo, Norway (O.A. Hassan);; Erasmus Medical Center, Rotterdam, the Netherlands (M.P.G. Koopmans);; University of Nyala, Nyala, Sudan (M.H. Ismail)

**Keywords:** Middle East respiratory syndrome coronavirus, MERS-CoV, zoonoses, One Health, preparedness and response, surveillance, policy, Gulf Cooperation Council, Middle East, viruses

## Abstract

In 2015, a One Health Working Group was established in Qatar to conduct a survey in the Gulf Cooperation Council countries, Egypt, and Jordan to monitor preparedness of public health and veterinary health authorities in response to the Middle East respiratory syndrome coronavirus epidemic. All but 1 country indicated they established joint One Health policy teams for investigation and response. However, the response to the questionnaires was largely limited to veterinary authorities. Critical barriers and limitations were identified. National and regional leaders, policy makers, and stakeholders should be prompted to advocate and enhance adoption of the One Health framework to mitigate the risk for Middle East respiratory syndrome and other emerging zoonotic diseases.

Human infections with Middle East respiratory syndrome coronavirus (MERS-CoV) continue to be reported from the Arabian Peninsula and the Middle East after the September 2012 World Health Organization (WHO) notification of 640 deaths from 2,040 laboratory-confirmed cases (*1*). Although typical symptoms of MERS-CoV infection include fever, cough, and labored breathing, pneumonia and diarrhea also were reported. Asymptomatic persons with laboratory-confirmed cases were observed as well (*2*). Saudi Arabia, the first country to report a confirmed MERS-CoV case, has had the most reported cases. Studies in Qatar and Saudi Arabia established the link between MERS-CoV and dromedary camels (*1*).

Camels are valued animals in arid and semiarid regions (*3*), where they serve as a basic source of milk and meat (*4*). The trading of camels and camel meat is an important source of income (*5*). In addition to the use of camels for food production, camels are popular for sport competition and beauty championships, which has led to formation of special camel institutions in some Arabian countries, including camel supreme councils and camel hospitals.

With the MERS-CoV outbreak as an emerging threat, the public health response included the possible role of camels in collaborative work with veterinary authorities to control and prevent the disease. Uncertainties about MERS-CoV transmission modes, coupled with growing evidence of the potential role of camels in disease dissemination, made this first trial of a One Health response challenging. A proper One Health response to a zoonotic disease requires several elements, including political support, appreciable preparedness and response plans, a joint vision on epidemiologic surveillance for MERS-CoV and zoonotic diseases in general, joint use of laboratory diagnostic capabilities, funding, and means for crisis communication and health education.

In Qatar, led by the Supreme Council of Health, a multidisciplinary team was established in 2014 once the zoonotic origin of the disease became evident. To discuss the challenges encountered during the MERS-CoV outbreak, and as part of international efforts to advance the adoption of the One Health approach to address health risks at the animal–human–environment interfaces (*6*), together with the Food and Agriculture Organization of the United Nations (FAO), Qatar organized in April 2015 a regional workshop in collaboration with the World Organisation for Animal Health (OIE) and WHO about the application of the One Health approach to MERS-CoV (*7*). Countries from the Gulf Cooperation Council (GCC; Saudi Arabia, Qatar, Kuwait, United Arab Emirates, Bahrain, and Oman), Egypt, and Jordan were represented in this workshop, along with delegates from FAO, OIE, WHO, the US Centers for Disease Control and Prevention (CDC; Atlanta, GA, USA), Erasmus Medical Center (Rotterdam, the Netherlands), the University of Hong Kong, and several other international experts.

To gauge a preliminary understanding about the extent to which the involved countries were using a One Health approach and how it was translated in government policies and practices, the One Health Working Group conducted a survey before the workshop. The findings were aggregated, presented, and discussed before the entire audience of the workshop.

## Methods

We designed the study based on guidance and references of the One Health approach established in documents issued by FAO, OIE, WHO (*6*), and CDC (*8*); meeting reports (*9*,*10*); and policy documentation (*11*). A questionnaire was drafted to answer queries about policies and structures governing control of zoonotic diseases in general and MERS-CoV in particular (Table 1). We shared the questionnaire with public health and veterinary authorities in charge of surveillance and control of MERS-CoV in all GCC countries, Egypt, and Jordan 1 week before the workshop. The questionnaire also included open-ended questions permitting comments. Results were analyzed and interpreted using an Excel spreadsheet (Microsoft, https://www.microsoft.com). The core of the questions and the relevant results scores are shown in Table 2. Results were presented and discussed before the survey participants and audience of the workshop and approved by the joint scientific committee of the workshop. Decision for dissemination followed consent of all of the survey participants.

**Table 1:** Domains of the questionnaire of the survey on the implementation of One Health for MERS-CoV preparedness and control in Gulf Cooperation Council and Middle East countries, 2015*

Principle	Title
1	Leadership and Coordination
2	Policies and Drivers of MERS-CoV Management
3	Preparedness and Response Plans
4	Epidemiologic Surveillance System
5	Laboratory Diagnostic Capacities
6	Crisis Communication and Health Education
7	The One Health Approach Operationalization Challenges

*MERS-CoV, Middle East respiratory syndrome coronavirus.

**Table 2:** Outcomes of survey questionnaire on the implementation of One Health for MERS-CoV preparedness and control in Gulf Cooperation Council and Middle East countries, 2015*

Domain	Subdomain	Response	
		Yes	No
Leadership and coordination	A. Existence of a dedicated MERS-CoV committee in surveyed institutions†	7	0
	B. The committee is meeting on regular basis†	6	1
	C. Participation of stakeholders in a joint committee or advisory board dealing with MERS-CoV at the national level†	5	2
	D. Activation of emergency supreme committee for MERS-CoV at the state level‡	2	4
Policies and drivers of MERS-CoV management	A. Existence of a document ascribing policy, roles, and responsibilities of committee’s stakeholders†	5	2
	B. The document describes the chain of command‡	4	2
	C. Joint committee responsibility for preparedness and response to MERS-CoV†	5	2
Preparedness and response plans	A. National plans for preparedness and response to MERS-CoV†	6	1
	B. Participation of stakeholders in preparation of national plans for preparedness and response to MERS-CoV‡	2	4
	C. Adequate budget allocation†	2	3
Epidemiologic surveillance system of MERS-CoV	A. Program of epidemiologic surveillance in humans‡	6	0
	B. Program of epidemiologic surveillance in animals‡	5	1
	C. Participation of animal breeders in MERS-CoV epidemiologic surveillance‡	3	3
	D. Joint or integrated surveillance program for MERS-CoV†	4	2
	E. MERS joint field investigation team†	5	2
	F. Field investigation joint team training‡	2	2
	G. Research program(s) for MERS-CoV†	2	4
Laboratory diagnostic capacities‡	A. Public Health Reference Laboratory	4	2
	B. Veterinary Reference Laboratory		
Crisis communication and Health education	A. Strategies and plans for information, crisis communication, and health education on MERS-CoV†	5	2
	B. MERS-CoV communication cooperation and coordination‡	5	1
	C. Joint implementation of MERS-CoV awareness and health education activities‡	3	3

*MERS-CoV, Middle East respiratory syndrome coronavirus. †Statistical analysis was performed by institution. ‡Statistical analysis was performed by country.

## Results

We surveyed 16 authorized government institutions representing 8 countries. Two countries did not respond. Seven (43%) institutions from 6 (75%) countries responded to the questionnaire. Six (85%) of 7 responding institutions were veterinary authorities. Except for 1 country, no public health authorities responded to the questionnaire.

### Leadership and Coordination

The 6 responding countries reported the existence of a joint veterinary and public health MERS-CoV committee (Table 2). Six institutions confirmed meeting on a regular basis. Five institutions from 4 countries reported having joint committees encompassing public health, veterinary services, municipalities, and research authorities. Two countries had an active emergency supreme committee at the national level addressing MERS-CoV crisis and threat.

### Policies and Drivers of MERS-CoV Management

Five institutions from 4 countries reported the presence of national documents detailing entitled authorities, policies, roles, commands, and responsibilities for stakeholders involved in MERS-CoV management. The same 5 institutions reported having a joint public health–veterinary authority committee responsible for preparedness and response to MERS-CoV following the standardized procedures developed by FAO and WHO.

### Preparedness and Response Plans

Six institutions from 5 countries had national early preparedness and response to MERS-CoV plans. Four of these institutions had clearly defined roles and responsibilities for each of the involved authorities (public health authority, animal health authority, environment authority, and others) during MERS-CoV threat or outbreaks. Only 2 (33%) countries had involved the major stakeholders (public health and animal health authorities) in the process of preparing a national plan for preparedness and response to MERS-CoV. Of the 7 institutions that answered the questionnaire, 2 reported adequate funding to address MERS-CoV, 3 denied adequacy, and the remaining 2 did not respond. Two institutions from 1 country did not agree on funding questions.

### Joint Epidemiologic Surveillance of Zoonotic Pathogens

All but 1 country reported having established a MERS-CoV epidemiologic surveillance program investigating vulnerable animals, camel owners, camel workers, breeders and keepers, slaughterhouse workers, and veterinary and medical personnel and sharing data with counterparts. Three countries reported participation of animal breeders; the other 3 reported the contrary. Four institutions from 4 GCC countries reported the existence of a joint epidemiologic surveillance program enabling outbreak investigation and sharing of reports and results. Two institutions reported lacking the joint surveillance, and 1 did not respond.

In 4 countries, 5 of 7 institutions indicated the presence of a joint public health–veterinary authority field investigation team and that MERS-CoV was jointly investigated. Two of the 4 countries organized an epidemiologic and disease control training course for the joint investigation team. Two countries initiated research programs in response to the outbreak.

### Joint Laboratory Diagnostic Capabilities

In 4 countries, national reference laboratories were established and identified to provide diagnostic services for human and animal MERS-CoV infection. The 4 countries reported national collaboration encompassing laboratory services, joint MERS-CoV diagnosis training, specimen shipping, and competency testing. Regionally, 2 GCC countries reported joint laboratory processing for MERS-CoV in camel samples. Three GCC countries reported joint activities with the Netherlands, Hong Kong, Germany, CDC, and the UK reference laboratories to fulfill international diagnostic and research requirements.

### Crisis Communication and Health Education

Five of 7 responding institutions from 4 countries reported having MERS-CoV crisis communication and health education strategic plans stating that the key stakeholders were involved in plans development. The 2 remaining countries either did not include these strategies in their national plans or were not aware of inclusion of these strategies.

Six responding countries reported providing MERS-CoV communication coordination mechanisms between public health and veterinary authorities covering awareness and health education. One country reported some conflicting messages between the 2 authorities. Three of the responding countries reported collaboration and implementation of awareness and health education issues during the MERS-CoV epidemic. In all but 1 country, camel breeders did not participate in the campaign.

### The One Health Approach Operationalization Challenges

Four of the 6 responding countries reported operational challenges encountered with adoption of the One Health approach. These challenges included lack of reliable and specialized diagnostic laboratories in the region, incapacity of the existing laboratories to yield MERS-CoV diagnostic services, and lack of skilled personnel tasked to investigate zoonotic cases. Other reported key challenges were misunderstanding of the One Health concept; conflicting priorities and plans; dearth of budgets allocated to meet MERS-CoV technical needs in terms of surveillance, diagnosis, control, and research; lack of skilled personnel on communication and health education; and the denial of camel breeders.

## Discussion

Because of the global increase in zoonotic threats, the importance of the One Health approach has also increased, along with the need to establish effective mechanisms for collaboration to address threats at the human–animal–environment interface (*6*,*8*,*12*,*13*). Affected by the United Nations agencies, several countries, particularly those challenged by zoonotic events, began initiating their One Health platforms and programs to enhance their capacities to manage zoonotic diseases (*10*,*11*,*14*–*16*). However, these efforts always faced many challenges.

To enable sufficient internal deliberations and ensure One Health quality and consensus-based responses, we shared the survey questionnaire with the relevant authorized health and veterinary institutions. However, the first hindrance was the response by only 44% of surveyed institutions, a fact that limited a comprehensive analysis of the outcomes. This low response rate could be attributed to poor leadership and to limited conceptual awareness about the One Health approach (*16*,*17*). This finding is sustained by our observation that there was a discrepancy understanding the One Health approach. Although the term is familiar among veterinarians, it is not among their health counterparts, a considerable drawback to implementing the approach. A high-capacity endeavor is needed advocating the health sector to deal with the One Health approach in the future.

Most of the GCC countries, including those with high MERS-CoV incidence, have adopted an epidemic control policy, indicating that the One Health approach was either partially embraced or totally overlooked. This finding was demonstrated by the fact that only 2 responding countries reported veterinary health authorities partnership formulating national preparedness and response plans. As a result, the quality of data collected in response to an outbreak remains questionable.

The lack of budget to support MERS-CoV control programs revealed by the survey questionnaire and the consequent workshop discussions emphasizes crucial points in the implementation of a successful One Health approach. One explanation may be that the cost for a proper One Health response had been underestimated. However, the disproportionate distribution of the available budget raised by the delegates might further explain the lack of integrated response. For instance, although most surveyed countries had established MERS-CoV epidemiologic investigation teams, only 50% of these teams react jointly. At the level of diagnostics, national laboratories in 66% of the countries managing and diagnosing MERS-CoV outbreaks had collaboration between medical and veterinary response, and several teamed up with international reference laboratories, which was considered a positive step toward diagnostic efficiency and cooperation. However, because MERS-CoV is a GCC home-country infection, the in-country diagnostic capacity was expected to be adequate.

When discussing crisis communication and health education, the core persistent barrier to embracing One Health seemed to be the prevalent denial of the camel owners that camels could be a potential source of MERS-CoV. Because of the highly influential role of camel owners among the communal sectors of most of the surveyed countries, involvement of these sectors to combat emerging zoonotic diseases is essential (*18*,*19*). However, because most local communities tend to react forcibly toward emerging infectious diseases (*20*), the investigators could neither judge this factor nor its effect in curbing the policy makers bolstering the One Health approach (*21*). Anticipating such socioeconomic risk factors, involvement of social scientists to resolve this barrier might help (*22*) facilitate community buy-in of One Health.

The survey results appear to show that respondents did not benefit much from the lessons learned during the last influenza A(H1N1) outbreak (*23*). The variation in the nature of MERS-CoV epidemiology among the countries—handled as a human-associated infection in some, a human–camel infection in others, and an unnoticed inapparent camel infection in others (*2*)—has imbalanced the magnitude of response among healthcare and veterinary sector authorities, a situation negatively affecting the application goals.

Given that the One Health approach is increasingly recognized internationally as an effective trend for managing emerging diseases at the human–animal–environment interface (*10*,*11*,*18*), the key barrier fostering the One Health approach at the national level suggested by this study seems to be the relative lack of political will. Based on the experience gained in addressing MERS-CoV at the human–animal interface, this lack of will could further be responsible for the poor sectoral response to the surveillance questionnaire. Although in Qatar, MERS-CoV was addressed through a One Health approach from the start (*24*), much remains to be done nationally, particularly at policy-making level. The foundation of a permanent interministerial committee might be a key step to raise awareness of leaders and policy makers using the concept and to determine the importance of the One Health approach. Creation of a supreme coordinating crisis communication committee is an important element to build zoonosis control and prevention capacities. A unified funding policy is a good incentive encouraging alleviation of the financial obligations accompanying One Health, expected to ease launching of joint investigations, intensive health educational sessions, epidemiologic surveillance programs, and joint seminars and workshops. Sharing of laboratory diagnostic research facilities, diagnostic protocols, and application of proficiency testing would help build experience and improve quality results. Joint routine veterinary health services programs application and adoption of compensation policy with continuous health education and extension programs might turn animal owners and other social stakeholders onto One Health.

The ratification of establishing a regional GCC center for infection control (*25*) to help develop unified standard and integrative guidelines to control zoonoses might help sustain the One Health approach. However, whether the current political situation might compromise the hope created by the previously promised political commitment to collaborate and allocate funds after the recent emergence of avian influenza A(H5N1) (*26*) remains questionable.
